# Population plasma and urine pharmacokinetics and the probability of target attainment of fosfomycin in healthy male volunteers

**DOI:** 10.1007/s00228-023-03477-5

**Published:** 2023-04-15

**Authors:** Angela Elma Edwina, Birgit C. P. Koch, Anouk E. Muller, Valentin al Jalali, Peter Matzneller, Markus Zeitlinger, Sebastiaan D. T. Sassen

**Affiliations:** 1 Department of Hospital Pharmacy, Erasmus University Medical Center, Rotterdam, The Netherlands; 2 Rotterdam Clinical Pharmacometrics Group, Rotterdam, The Netherlands; 3 Gerontology and Geriatrics Unit, Department of Public Health and Primary Care, KU Leuven - University of Leuven, Leuven, Belgium; 4 Department of Medical Microbiology and Infectious Diseases, Erasmus University Medical Center, Rotterdam, The Netherlands; 5 Department of Medical Microbiology, Haaglanden Medical Center, The Hague, The Netherlands; 6 Department of Clinical Pharmacology, Medical University of Vienna, Vienna, Austria; 7 Service of Rheumatology, Hospital of Merano, South Tyrol Health System ASDAA-SABES, South Tyrol, Italy

**Keywords:** Population pharmacokinetics, Fosfomycin, Antimicrobial, *Escherichia coli*

## Abstract

**Purpose:**

A population pharmacokinetic model of fosfomycin was developed in healthy volunteers after intravenous administration, and different dosing regimens were evaluated in terms of the probability of target attainment for *Escherichia coli* using both plasma and urinary pharmacokinetic/pharmacodynamic targets.

**Methods:**

Eight healthy men received fosfomycin as both intermittent 8 g q8h and continuous infusion 1 g/h with a loading dose of 8 g in a crossover study design. Dense sampling was conducted during both regimens. Population pharmacokinetic modelling was performed using NONMEM. Monte Carlo simulations were conducted to evaluate the Probability of Target Attainment (PTA) of different dosing regimens using bactericidal (AUC_24h_/MIC of 83 and 75%*T*_>MIC_) and bacteriostatic (AUC_24h_/MIC of 25) plasma targets and bacteriostatic (AUC_24h_/MIC of 3994) urine target.

**Results:**

A total of 176 plasma and 86 urine samples were available for PK analysis. A two-compartment model with a urine compartment best described the data. Glomerular filtration rate (GFR) showed a significant correlation with renal clearance and was implemented in the final model. Simulation results show that the dose of 4 g q8h reached 100% of PTA using bactericidal and bacteriostatic targets for MIC up to 16 mg/L.

**Conclusion:**

For the clinical breakpoint of 32 mg/L, the standard dosing regimen (4 g q8h) might not be sufficient to reach the bactericidal target. Higher dosing of 8 g q8h as an intermittent infusion or 0.75 g/h as a continuous infusion might be required. Continuous infusion resulted in better attainment of the %*T*_>MIC_ target than intermittent infusion.

**Supplementary Information:**

The online version contains supplementary material available at 10.1007/s00228-023-03477-5.

## Introduction

In recent years, the increased prevalence of multi-drug-resistant, extensively drug-resistant, and pan-drug-resistant bacteria is a critical threat to global health [1]. Treating infections caused by resistant bacteria poses significant challenges due to the dwindling number of effective antibiotics and the lack of new antibiotic development [2]. To tackle this problem, other strategies, especially the use of older antibiotics, have to be considered [3].

One of the older antibiotics is fosfomycin, a cell wall synthesis inhibitor with a broad-spectrum antimicrobial activity that was first developed in 1969 [4, 5]. Lately, because of its activity against extended-spectrum beta-lactamase- and carbapenemase-producing Enterobacterales, fosfomycin has been acknowledged as a valid treatment option for infections caused by difficult-to-treat Enterobacterales [6–9]. In general, an intravenous formulation of fosfomycin (i.e. fosfomycin disodium) is prescribed to treat more severe and complicated infections [10]. The approved dosing schedules for intravenous fosfomycin are 12–24 g/day, which can be given in 2 or 3 dosages [11]. In addition, the dosing of 16–24 g in 3 or 4 divided dosages is needed to treat bacterial meningitis [11]. The European Committee on Antimicrobial Susceptibility and Testing (EUCAST) recommends dosing regimens of 16–18 g of fosfomycin/day, divided into 3–4 dosages [13]. However, more studies are urgently needed to determine the optimal dose regimen to achieve favourable efficacy while avoiding adverse effects, such as a rash, peripheral phlebitis, hypokalaemia, and gastrointestinal disorders [14, 15].

The link between the pharmacokinetics (PK) and pharmacodynamics (PD) of fosfomycin provides insight into the relationship between dose, concentration, and effect [16]. Additionally, both plasma and urine concentration profiles are essential as fosfomycin is used to treat urinary tract infections (UTI) and systemic infections [11]. Although a number of studies on the population PK of fosfomycin have been conducted, most of these focused on plasma PK. Consequently, urinary PK is not fully understood [17–20]. Only a limited number of PK studies have evaluated plasma and urine samples simultaneously in healthy volunteers, utilising conventional PK analyses that did not include a covariate analysis [21–23]. One of the most recent fosfomycin PK analyses was studied in healthy volunteers, and it was the first PK data on fosfomycin continuous infusion [15]. However, the assessment of PK/PD target attainment following different dosing regimens based on a population PK model has not been done yet. In this study, a combined plasma and urine population PK model of intravenous fosfomycin was developed from published data of healthy male volunteers [15] and used to explain fosfomycin disposition and subject characteristics’ effect on PK parameters. Subsequently, Monte Carlo simulations were conducted to evaluate different dosing regimens and different values of the significant covariate(s) for *Escherichia coli* infection treatment with regard to reaching pharmacokinetics/pharmacodynamics (PK/PD) targets in both plasma and urine as a surrogate marker for efficacy.

## Materials and methods

### Study design and population

This analysis is based on the published data of a prospective, open-label, single-centre, randomised crossover study that was performed at the Department of Clinical Pharmacology at Medical University Vienna [15]. Data were obtained from eight healthy male volunteers. The study protocols were approved by the local ethics committee and the Austrian Agency for Health and Food Safety (EudraCT registration number 2018–000,653-45). Details on recruitment, inclusion–exclusion criteria, and informed consent have been described in a previous study by al Jalali et al. [15].

### Study procedures and sampling

Subjects were randomised to receive fosfomycin first as an intermittent infusion or a continuous infusion. After a washout period of at least 48 h, subjects received the second treatment regimen. An intermittent infusion of 8 g fosfomycin was administered over 30 min every 8 h for a total of three dosages. During the continuous infusion, a loading dose of 8 g fosfomycin was administered as an intermittent infusion over 30 min, followed by continuous infusion at a rate of 1 g/h over 18 h corresponding to 24 g fosfomycin/day.

Dense blood and urine sampling were conducted. For intermittent infusion, blood samples were taken before and after each intermittent infusion, additionally after the start of the third infusion, at the following time points: 1, 1.5, 2, 4, 5, 6, and 8 h. During continuous infusion, plasma sampling was performed before and at the end of the loading dose infusion. Subsequently, plasma samples were collected at 1, 3, 5, 8, 16, 17, and 18 h after the administration of the loading dose. Urine samples were collected during 24 h and 18 h for intermittent and continuous infusion, respectively, at 0–3 h, 3–8 h, 8–16 h, 16–18 h, 18–20 h, 20–22 h, and 22–24 h after the start.

Fosfomycin concentrations in plasma and urine samples were quantified using an ultra-performance liquid chromatography-tandem mass spectrometry (UPLC-MS/MS) method with hydrophilic interaction chromatography. The lower and upper limits of quantification (LOQ) of this assay are 0.75 and 375 mg/L, respectively. The measurement was conducted in the ISO 15189-accredited laboratory at the Department of Pharmacy, Erasmus MC, Rotterdam, the Netherlands [24].

### Pharmacokinetic analysis

Fosfomycin plasma and urine concentrations were analysed simultaneously using the nonlinear mixed effects modelling approach implemented in NONMEM® version 7.4 (Globomax LLC, Ellicott City, MD, USA) [25] using the first-order conditional estimates with interaction (FOCE-I) algorithm. Tools utilised to evaluate and visualise the model included R (version 4.0.1) [26], RStudio (version 1.3.959) [27], Xpose (version 4.7.1) [28], Perl-speaks-NONMEM [29], and Pirana (version 2.9.9) [29, 30].

The population PK model was developed with a stepwise approach. For the structural plasma PK model, one- and two-compartment models were explored, followed by the addition of a urine compartment. Analyses on separated regimens, including intermittent infusion or continuous infusion alone, were also conducted. Residual variability was evaluated using a proportional, additive, and combined error model. Inter-individual variability was tested using an exponential variance model. Model selection criteria were based on quantitative evaluations such as a decrease in the objective function value (OFV), the precision of the estimated PK parameters, and the condition number, which is an indicator of model stability and identifiability. A decrease in objective function value (OFV) of 3.84 units was considered a statistically significant improvement (*p* < 0.05 assuming chi-square distribution) for one degree of freedom. Additionally, graphical evaluations, including goodness-of-fit plots (GoF) and visual predictive checks (VPC), were used to assess the model’s performance. The robustness of the model parameters was evaluated using a nonparametric bootstrap procedure (*n* = 1000).

The final structural model was used for the covariate analysis. A covariate model building was performed with iterative forward inclusion at *p* < 0.05 and backward elimination at p < 0.01. The tested covariates were age, body weight, height, BMI, total protein, albumin, serum creatinine concentration (sCr), estimated glomerular filtration rate (eGFR) calculated with the Chronic Kidney Disease Epidemiology Collaboration equation (CKD-EPI) in millilitres per minute, eGFR calculated with the CKD-EPI formula normalised to 1.73 m^2^ of body surface area [31], and creatinine clearance (CrCl) estimated with the Cockroft-Gault formula [32]. All covariates were evaluated as continuous covariates and were centred around the median.

### PK/PD simulation and target attainment

Using the final population PK model, Monte Carlo simulations of 1000 virtual subjects were performed with NONMEM to determine PK/PD target attainment for various dosing regimens and different values of the significant covariate(s). The plasma PK/PD indices were chosen. These indices were most likely linked to the bacterial burden decrease by 1-log and net bacterial stasis for *E. coli*, which are AUC_24h_/MIC of 83 and AUC_24h_/MIC of 25 in a murine thigh model, respectively [33]. One-log kill was also observed at *T*_>MIC_ values of 52–100%, so the value of 75%*T*_>MIC_ was chosen as the time-dependent killing activity index [33]. Those targets in plasma are based on total concentration, as fosfomycin is a nonprotein-bound antibiotic [34]. For the target attainment in urine samples, the bacteriostasis PK/PD target of 3994 for the Enterobacteriaceae group was used as there is no specific target for *E. coli* [12]. The MIC values for *E. coli* were selected from the EUCAST data and ranged from 0.25 to 512 mg/L. This includes the Epidemiological Cut-off of 4 mg/L as well as the current EUCAST clinical breakpoint of 32 mg/L [12]. Common dosing regimens were simulated. The probability of target attainment (PTA) per dosing regimen was calculated as the percentage of simulated subjects achieving the PK/PD target after 24 h dosing. Additionally, simulations were performed for different values of the significant covariate(s).

## Results

### Subjects and samples

Subject characteristics are described in Table 1. Subjects’ body size, levels of protein and albumin, and kidney function were within the normal range. Only one subject was slightly underweight, and one subject had a mild reduction of kidney function. Dense sampling resulted in 22 plasma and 9–11 urine samples per patient corresponding to a total of 176 plasma and 86 urine samples. One nonzero plasma concentration (11.02 mg/L) after a washout period was excluded from analysis as there should be no drug detected in plasma considering the previous level was a lot lower without new drug administration prior to sample and the individual clearance. Six subjects after intermittent infusion and seven subjects after continuous infusion had cumulative amounts in urine that exceeded the total given dose. In the modified dataset, the exceeding amount was reduced to the maximum administered dosages of 24 g for intermittent infusion and 26 g for continuous infusion.

**Table 1:** Subject characteristics

**Characteristics**	**Median (range)**
Age (years)	36.5 (23-42)
Weight (kg)	83.5 (53–93)
Height (cm)	1.81 (1.71–1.96)
BMI (kg/m^2^)	24.18 (18.1–27.3)
sCr (mg/dL)	0.91 (0.79–1.19)
eGFR (mL/min)	119.63 (89–138)
CrCL (mL/min)	128 (97–151)
Albumin (g/dL)	44 (41.5–48.9)
Total protein (g/dL)	69.45 (62.8–73)

*BM* Body Mass Index, *sCr* serum creatinine, *eGFR* estimated Glomerular Filtration Rate calculated using CKD-EPI formula, *CrCL* creatinine clearance

### Population PK analysis

**Fig. 1:**
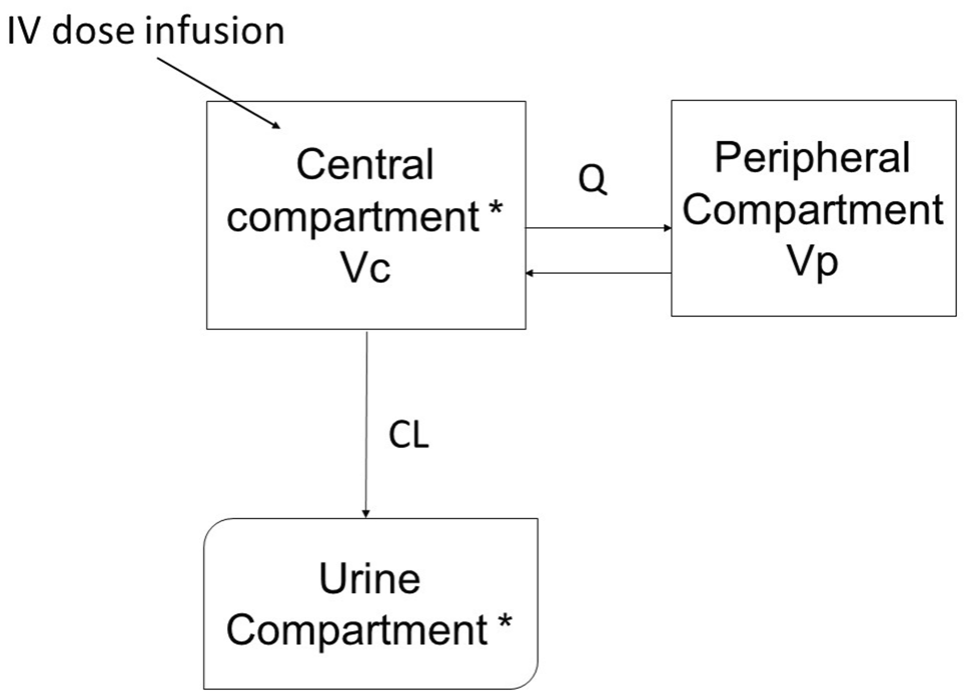
Schematic representation of population PK model for fosfomycin. *observation

A two-compartment model best described fosfomycin plasma and urine concentrations with an additional urine compartment (see Fig. 1) and inter-individual variability on renal clearance. Residual errors for both plasma and urine compartments were best described using proportional error models. After the covariate model was built, eGFR (CKD-EPI in mL/min), which had a significant positive correlation with clearance, was the only covariate included in the final model (*p* < 0.01, Fig. S2). The NONMEM code for the final model is provided in the Supplementary material. The model-building steps are also presented in Supplementary Table 4.

**Fig. 2:**
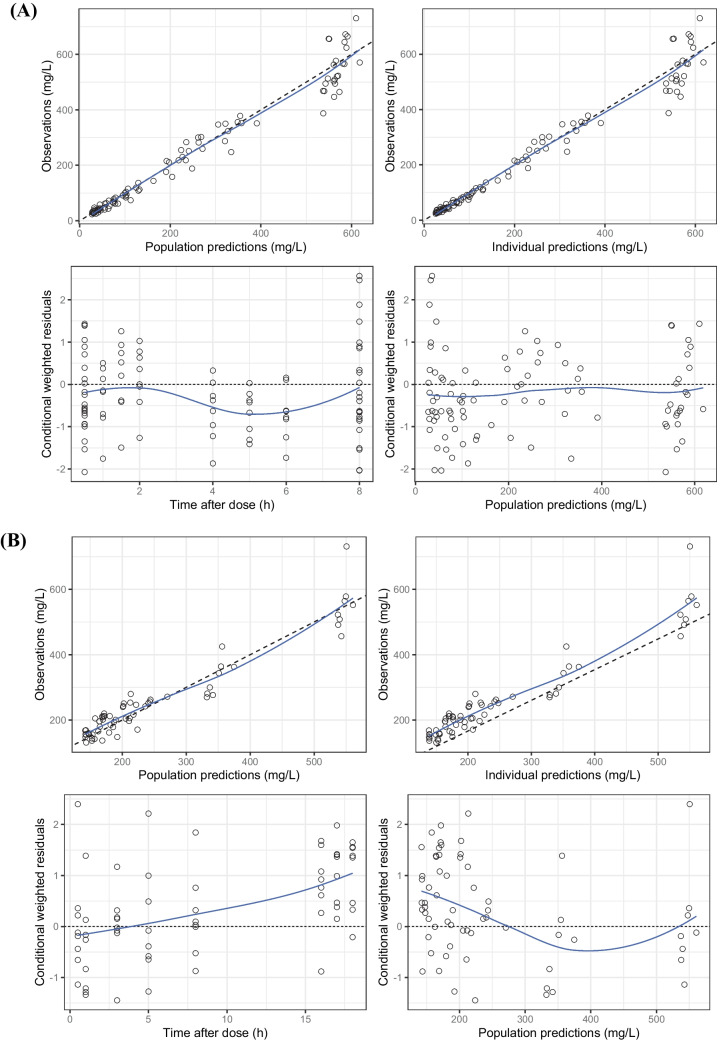

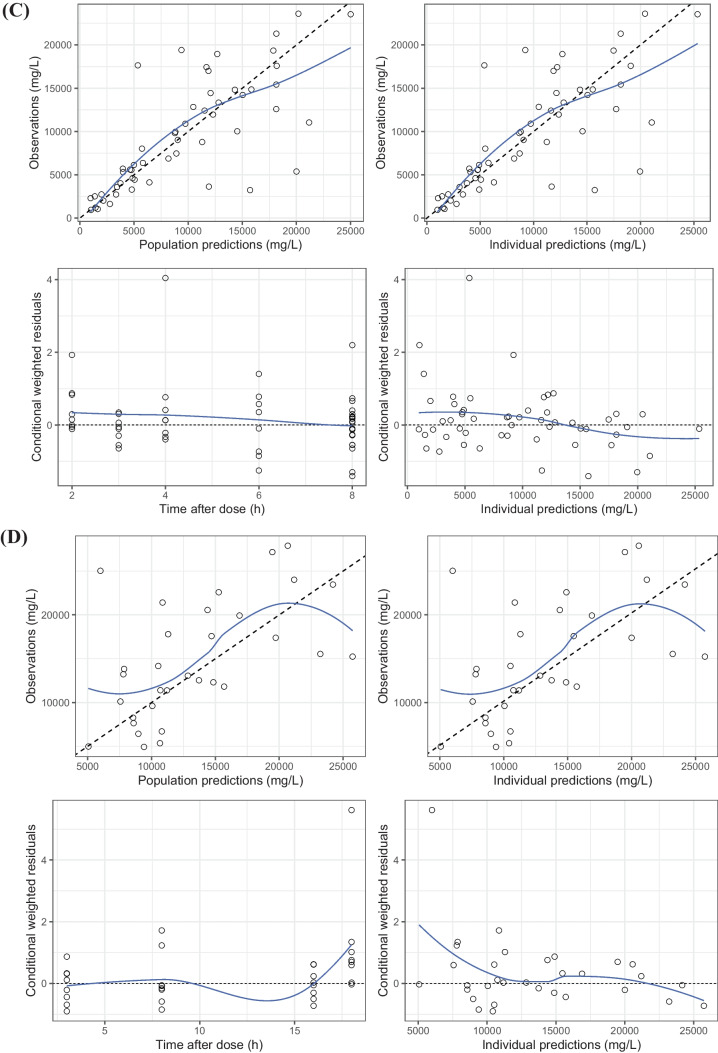
Goodness-of-fit plots of **A** plasma observation following intermittent infusion administration, **B** plasma observation following continuous infusion administration, **C** urine observation following intermittent infusion administration, and **D** urine observation following continuous infusion administration

**Fig. 3:**
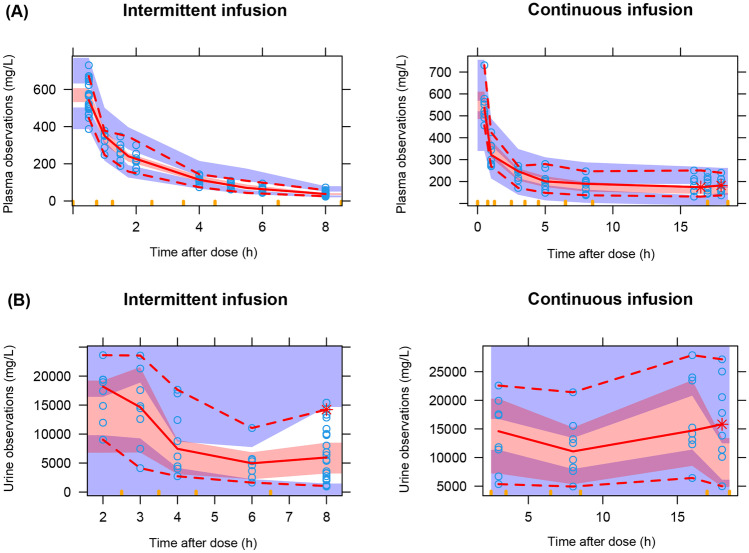
Visual predictive check for the fosfomycin pharmacokinetic final model with plasma (**A**) and urine (**B**) data. The solid red lines represent the median observed concentrations, and the shaded red areas show the simulation-based 95% VPC interval for the median. The red dashed lines represent the observed 5th and 95th percentiles, and the surrounding purple areas indicate the simulated 5% and 95% VPC intervals for the corresponding predicted percentiles

Overall, the model complies with the criteria set for the model with respect to precision, stability, and robustness. The goodness-of-fit plots demonstrate that the model predictions were in line with the observed plasma concentrations (see Fig. 2). The model can predict the urine concentrations, albeit with a larger variability compared to the plasma concentrations. The VPC (see Fig. 3) showed that the model described the observed data well. A high condition number (8.5e^7^) after the addition of a covariate was identified, which was caused by an overfitting issue as the sample size was small and the population was homogenous. Although the condition number was high for the final model, very different initial estimates resulted in the same parameter estimates, indicating that the model is stable and reliable. Therefore, the covariate model with a high condition number was chosen as the final model. The final parameter estimates and bootstrap results are detailed in Table 2.

**Table 2:** PK parameters and bootstrap

**Final model**			**Bootstrap results**	**SIR results**
**Parameters**	**Parameter estimates**	**RSE % (shrinkage %)**	**95% CI**	**95% CI**
CL (L/h)	CL_pop_* (eGFR/120)^β^	–	–	
CL_pop_	6.34	3.3	5.992–6.766	6.045–6.695
*β*	0.774	14.2	0.478–1.071	0.608–0.981
*Q (L/h)*	11.8	10.9	9.251–14.275	9.485–13.412
Vc (L)	10.2	7.7	8.731–11.689	9.089–11.558
Vp (L)	9.74	6.2	8.852–10.943	8.923–10.832
Interindividual variability of CL (%CV)	4.8	34.8 (4%)	1.05–7.26	2.32–7.69
Proportional error in plasma (%CV)	38.17	4.5	33.85–41	34.91–41.01
Proportional error in urine (%CV)	87.15	9.3	64.42–104.96	69.38–105.39

*CL* clearance, *CLR*_*pop*_ population renal clearance, *β* the effect of eGFR on renal clearance, *Q* intercompartmental clearance, *Vc* central volume of distribution, *Vp* peripheral volume of distribution

### PK/PD simulation

**Fig. 4:**
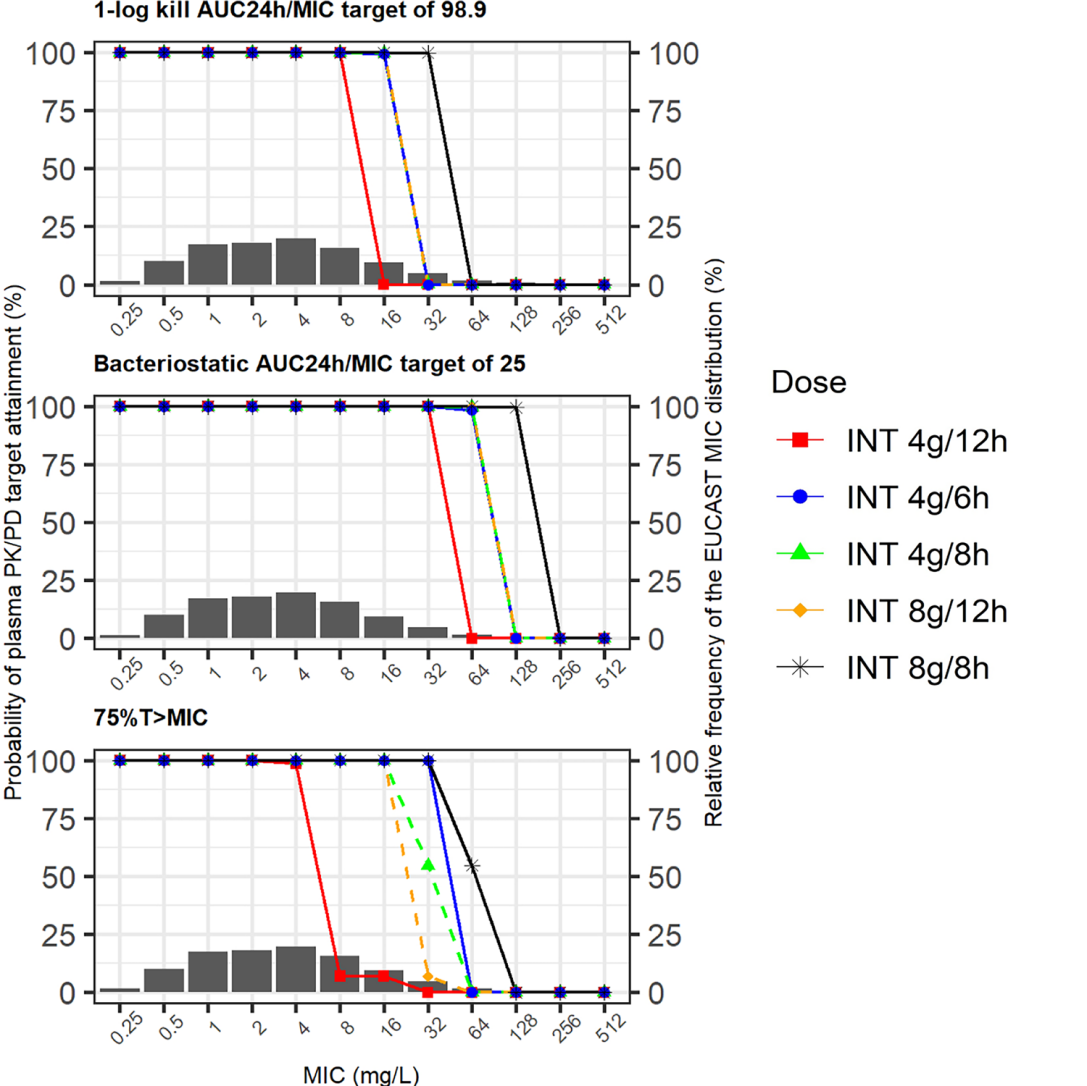
Probability of target attainment (PTA) for various fosfomycin intermittent infusion (over 30 min infusion) dosing regimens to reach plasma PK/PD targets: A AUC_24h_/MIC of 83, B AUC_24h_/MIC of 25, and C 75%*T*_>MIC_. The bar plots represent the relative frequency of the EUCAST MIC distribution (right *y*-axis) across different values of MIC in the *x*-axis

**Fig. 5:**
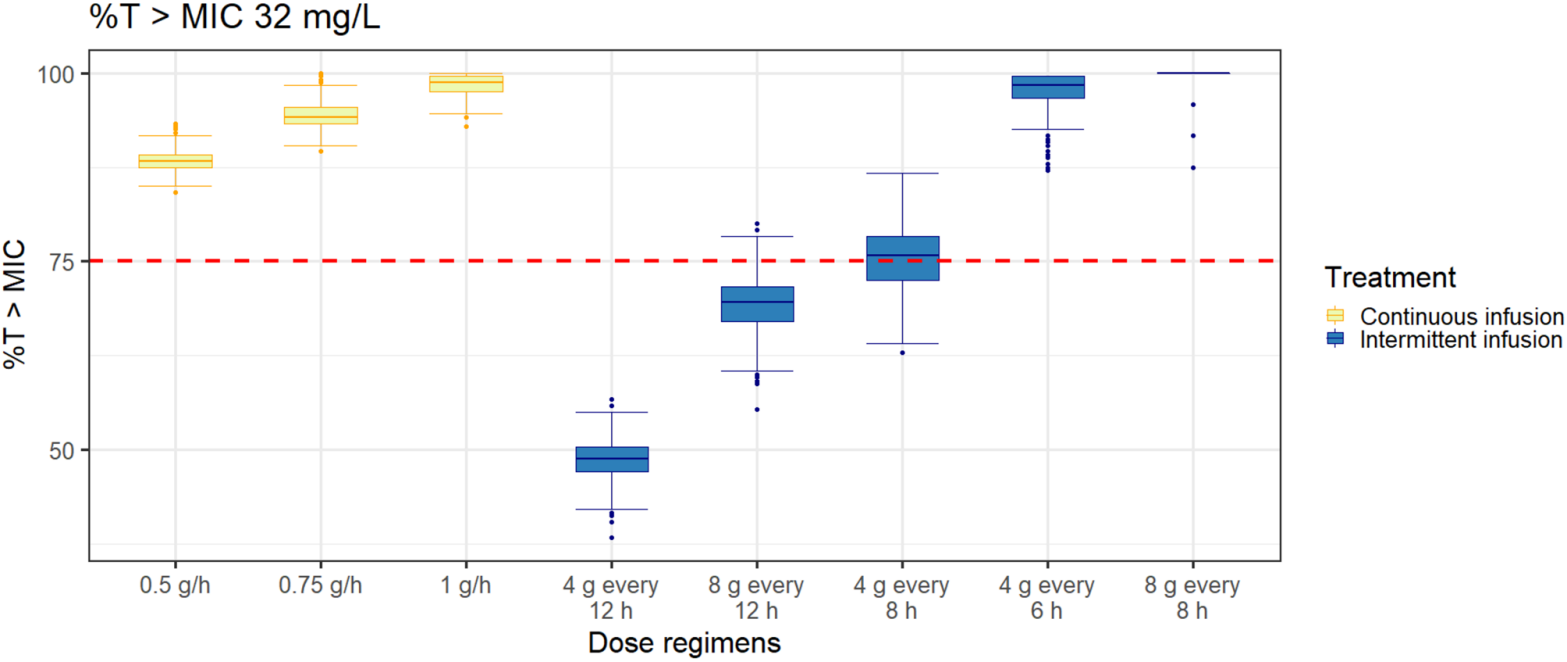
Plasma %*T*_>MIC_ attainment for MIC 32 mg/L of various dose regimens as a continuous infusion (yellow boxplots) and intermittent infusion (blue boxplots) in simulated subjects (*n* = 1000 iterations). The red dashed line is the target of 75%*T*_>MIC_

Figure 4 shows the PTA for various simulated intermittent infusion dosing regimens. The more detailed PTA results are presented in the Supplementary Tables. All dosing regimens achieved a 1-log kill bacterial reduction target in plasma (AUC_24h_/MIC 83) for MIC ≤ 8 mg/L, while for MIC > 32 mg/L, no dosing regimen attained the target. Using a bacteriostatic target (AUC_24h_/MIC 25), all dosing regimens achieved 100% of PTA to kill bacteria with MIC ≤ 32 mg/L, and intermittent infusion of 8 g every 8 h attained 100% of PTA for MIC up to 128 mg/L. Targeting 75%*T*_>MIC_, all continuous infusion regimens, 4 g every 6 h and 8 g every 8 h as intermittent infusion, reached 100% of PTA for MIC ≤ 32 mg/L (Fig. 5).

**Fig. 6:**
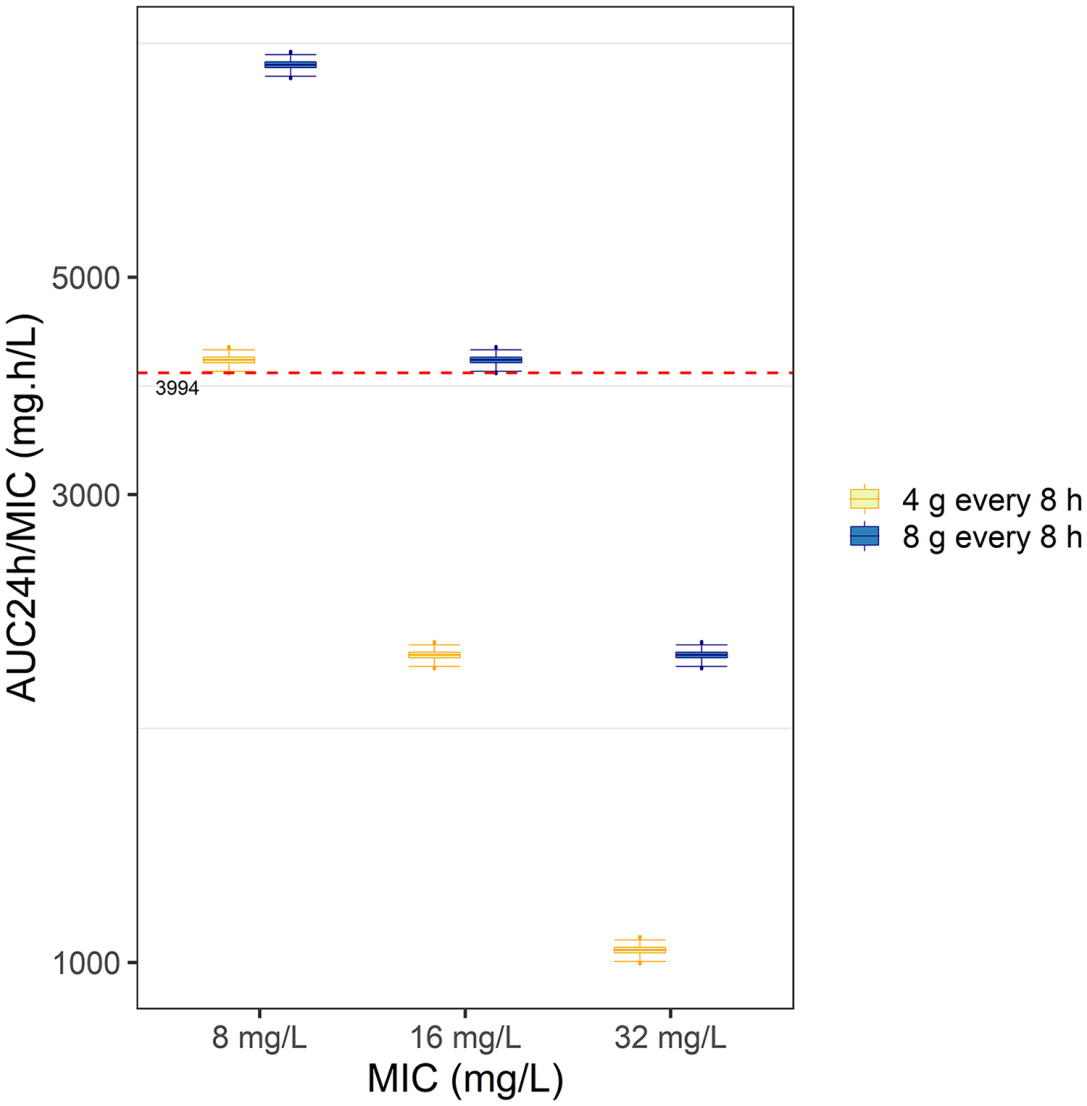
PTA targeting urinary AUC_24h_/MIC for MIC 8, 16, and 32 mg/L following a fosfomycin dose of 8 g every 8 h (blue boxplots) and 4 g every 8 h (yellow boxplots) as an intermittent infusion in simulated subjects. The red dashed line is the target of AUC_24h_/MIC of 3994

In addition, a simulation to evaluate urinary PTA was conducted using the target of AUC_24h_/MIC 3994 for various MIC values (see Fig. 6). Almost 100% of simulated subjects receiving 4 g fosfomycin every 8 h met the target for MIC 8 mg/L, but none of the subjects met the target for MIC 16 mg/L or higher. The target of MIC 16 mg/L could be achieved by administering 8 g of fosfomycin every 8 h. No simulated dose seems to reach the urinary target of MIC 32 mg/L or higher.

Simulations based on different eGFR values (90, 110, 120, and 140 mL/min) for a subject receiving 1 g/h continuous infusion and 8 g three times daily intermittent infusion were performed (see Fig. S1). The more detailed simulation results are presented in the Supplementary Tables. For MIC ≤ 32 mg/L, all simulated subjects with defined eGFR values attained the 1-log bacterial reduction target of AUC_24h_/MIC 83 following 8 g every 8 h of intermittent infusion and 1 g/h continuous infusion. Subjects with higher eGFR values had a lower probability of attaining targets than subjects with lower eGFR values.

### Plasma and urine fosfomycin exposure and target attainment

**Fig. 7:**
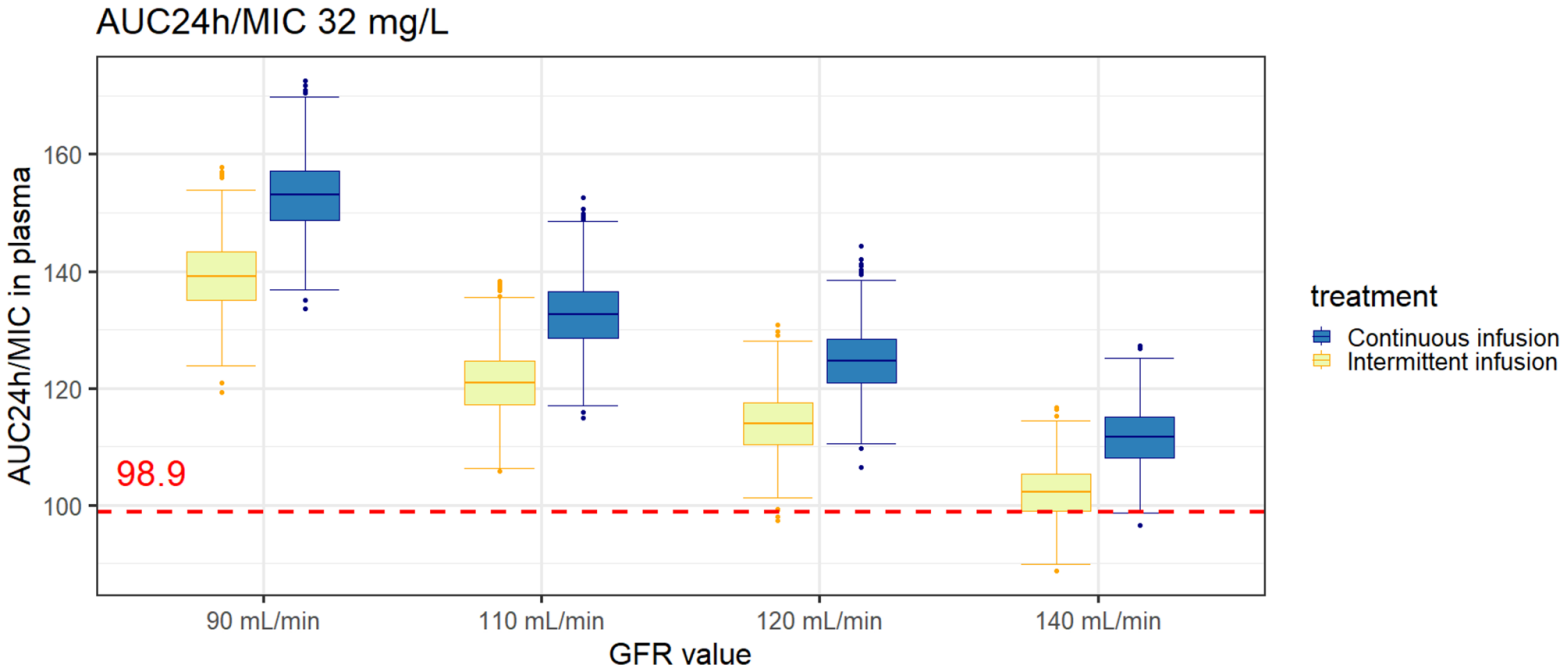
AUC_24h_/MIC plasma attainment for MIC 32 mg/L of different eGFR values as a continuous infusion of 1 g/h (yellow boxplots) and intermittent infusion (blue boxplots) of 8 g every 8 h in simulated subjects (*n* = 1000 iterations). The red dashed line is the 1-log bacterial reduction target of AUC_24h_/MIC of 83

**Fig. 8:**
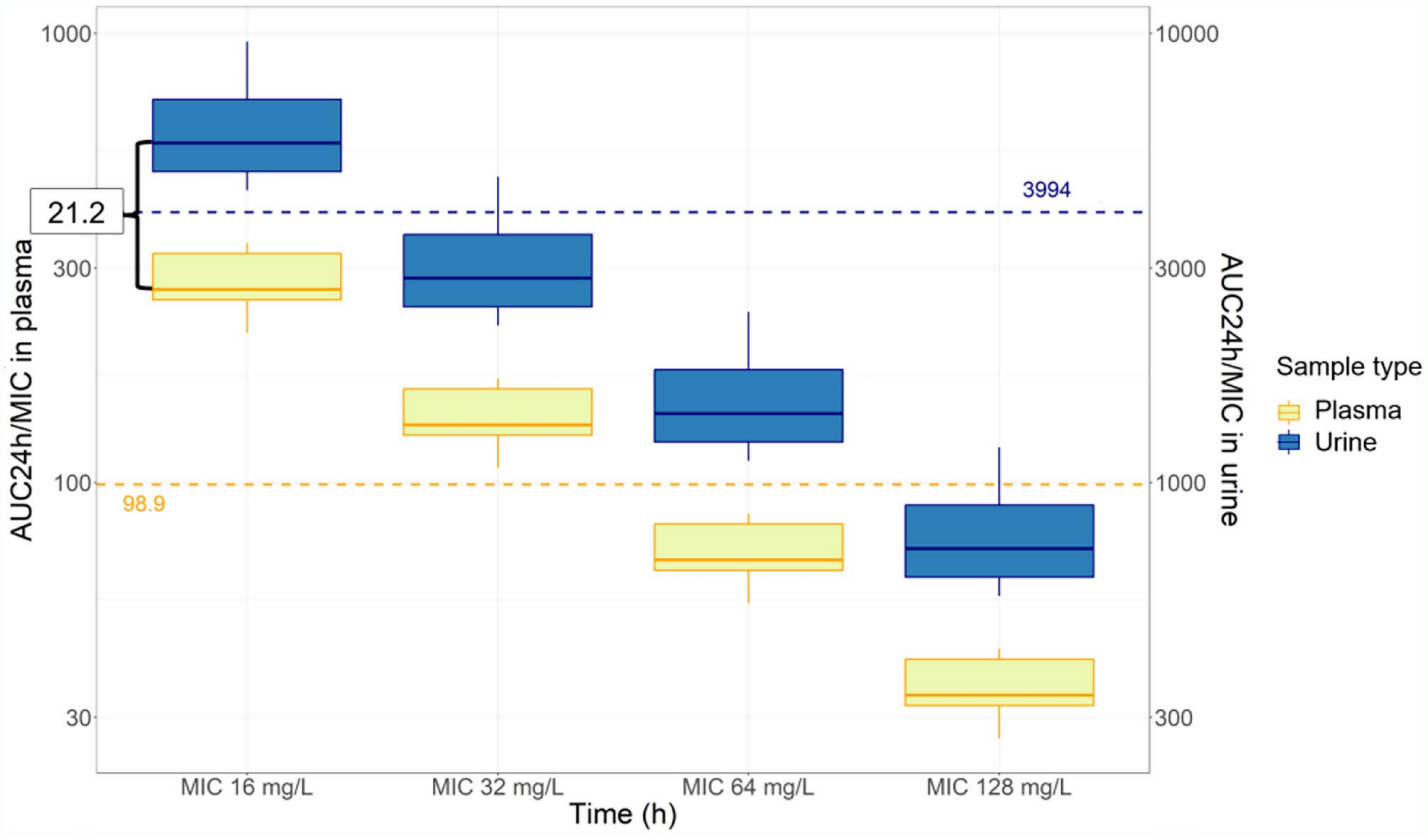
The fosfomycin plasma (blue boxplots) and urine (red boxplots) AUC_24h_/MIC in study participants following intermittent infusion administration of 8 g every 8 h. The blue dashed line indicates the AUC_24h_/MIC target in plasma of 83, and the red dashed line indicates the AUC_24h_/MIC target in the urine of 3994 based on EUCAST. The median ratio between urine and plasma AUC_24h_ was 21.2

Figure 7 shows plasma and urinary fosfomycin concentrations in healthy volunteers after intermittent and continuous infusion. Within 24 h, the cumulative urinary excretion achieved around 100% of the daily dose. Fosfomycin administration as a continuous infusion resulted in a constant urinary excretion rate. Meanwhile, rapid urinary rate excretion was observed after the third dose of intermittent infusion. Additionally, fosfomycin urinary exposure, or AUC_24h_, was approximately 21 times higher than plasma exposure in healthy volunteers (see Fig. 8).

### Toxicity analysis

The original article by al Jalali et al. reported thrombophlebitis events in two subjects during continuous infusion administration [15]. Those two subjects had relatively high maximum concentrations of 730.99 and 577.98 mg/L, which were above the mean maximum concentration of 550.41 mg/L in study participants (Supplementary Table 3).

## Discussion

Using plasma and urine samples, our study characterised the fosfomycin population PK and subject characteristics affecting the PK of fosfomycin, which serves as initial data for developing further nomograms for fosfomycin. The present finding suggests the sufficiency of intravenous fosfomycin standard dosing regimens of 4 g every 8 h as an intermittent infusion using the target of AUC_24h_/MIC 83 for 1-log bacterial reductions with a MIC of 4 mg/L, which is the current epidemiological cutoff values (ECOFF) of fosfomycin for *E. coli*. However, the ECOFF for fosfomycin is much lower than the EUCAST clinical breakpoint of 32 mg/L [12]. For the current clinical breakpoint of 32 mg/L, the currently recommended standard dosing regimen of 4 g every 8 h might not be sufficient, and a higher dosing of 8 g every 8 h as an intermittent infusion or 0.75 g/h as a continuous infusion might be required to reach the AUC_24h_/MIC 83 target.

Our results seem to be in line with the EUCAST current clinical breakpoint as no simulated dosing regimen is considered adequate to attain AUC_24h_/MIC target for the purpose of killing resistant bacteria (MIC > 32 mg/L). To exhibit the bacteriostatic effect of fosfomycin with the target of AUC_24h_/MIC 25 against *E. coli* with MIC 32 mg/L, 4 g every 12 h may be adequate. Thus, combining fosfomycin with other antibiotics should be considered as the main alternative to demonstrate the bactericidal effect against fosfomycin-resistant *E. coli* and to prevent resistance in general. A combination of fosfomycin and cefixime exhibited synergistic to kill resistant *E. coli* based on a previous in vitro study [36].

As concentration at the local site of infection is critical, simulations analysing the urine PK/PD target (AUC_24h_/MIC 3994) were performed. The present evaluation indicated that the standard daily dose schedule of intravenous fosfomycin (4 g every 8 h) can be used to treat urinary tract infections caused by susceptible *E. coli* with a MIC of 8 mg/L. However, it may not be effective against *E. coli* with a MIC of 16 mg/L meaning that if the dosing regimen attains the plasma target, the urinary target is not necessarily attained. This supports the results of the established in vitro dynamic bladder infection model, which also proved that *E. coli* was hardly killed with a MIC of 16 mg/L [35]. This result also suggests that the current breakpoint might be too high for the urine target of AUC_24h_/MIC 3994. Nevertheless, using only urine samples is generally not considered the best choice for therapeutic drug monitoring (TDM), since the collection of urine samples can be burdensome for some patients and thus may not be feasible in the majority of cases. Our study offers the feasibility of predicting fosfomycin exposure in urine by measuring plasma concentration and using a urine-plasma exposure ratio of 21,2. Yet, the link between urine and plasma concentration should be investigated more comprehensively to optimise the plasma PK/PD target that is strongly related to efficacy in the urinary tract.

The PK/PD index related to the time-killing activity of fosfomycin against *E. coli* remains disputed, as the target of %*T*_>MIC_ was less robust than the AUC/MIC target in an earlier in vivo study [33]. Yet, a previous in vitro pharmacodynamic study proved that three out of the five *E. coli* strains investigated in the study seemed to be more time-dependent and two others were concentration-dependent [37]. To treat *E. coli* strains that are more time-dependent, our simulation proposed continuous infusion rather than intermittent infusion since the concentration remains constantly above the MIC. This is in line with al Jalali et al.’s noncompartmental analysis demonstrating that fosfomycin as a continuous infusion resulted in an improvement in the attainment of PK/PD determinants [15]. Our simulation of PTA targeting 75%*T*_>MIC_ can be used as a direction to treat *E. coli*, and these results need to be validated in clinical studies.

Although fosfomycin is considered a safe drug and is generally well tolerated, possible fosfomycin toxicity was observed in this study. Two subjects experienced thrombophlebitis after continuous infusion administration [15]. A previous literature review and analysis of the reporting system database demonstrated that peripheral phlebitis was one of the most frequent adverse events of parenteral fosfomycin [14], which is in line with the result of the current study. Thrombophlebitis might be related to fosfomycin exposure, as these were observed in the two subjects with the highest maximum concentrations. Unfortunately, the evidence of fosfomycin toxicity, especially thrombophlebitis, and its relation to its exposure is lacking. No data on the threshold of fosfomycin toxicity are available.

It is known that fosfomycin is eliminated primarily by the kidneys, as a result, the correlation between eGFR and clearance of fosfomycin was expected, which was also the case in the previous studies showing kidney function as a significant covariate [22, 36, 37]. It is certainly assumed, but not confirmed by this analysis, that there is a need for dose reduction in patients with renal insufficiency based on eGFR calculated with the CKD-EPI formula. We tested the effect of kidney function on the fosfomycin PK using three different markers, including serum creatinine, eGFR calculated with the CKD-EPI formula, eGFR normalised to 1.73 of body surface area, and CrCl calculated with the Cockcroft–Gault equation. In this analysis, eGFR (mL/min) showed a strong correlation with CL and is considered a better kidney function marker than CrCL by Cockcroft–Gault, especially for some populations where it could underpredict. This finding is not in line with the product information, where the standard dosing regimen of fosfomycin is adjusted based on CrCl [11]. Therefore, to achieve a more optimal dosage, this model suggests adjusting dosing based on eGFR instead of CrCl.

The model showed a good ability to characterise fosfomycin PK based on model diagnostics and validation. Most other recent studies also confirmed that the two-compartment model was the best fit to describe the fosfomycin plasma PK [17–20]. The second exponential decay suggests a distribution into deeper tissue, which leads to a slower release into plasma. It has been reported that fosfomycin penetrates extensively into the interstitial fluid of soft tissues [38–40]. Parameter estimates calculated in this study were comparable with those previous studies in healthy volunteers [15, 21–23, 41, 42].

Despite the successful PK model development using plasma and urine samples, this study has potential limitations. The data are from healthy males, and extrapolation to other groups like severely ill patients and women is limited. The PTAs of fosfomycin in patients, especially those with impaired renal function, can be higher than in healthy volunteers, and thus the dosing recommendation needs to be reduced. However, the dosing recommendation for patients with an eGFR of < 90 mL/min was not evaluated in this study. Therefore, a future external validation study should be conducted to assess the model’s fit for the target (extrapolated) population with a wider range of eGFR values, beyond those observed in our study participants. Another limitation is related to the urine data, which was modelled with high residual variability. The recovery in urine was over 100% in six subjects, which was possibly due to errors in the urine collections, which were done manually, or due to small assay errors. Fortunately, both the original and modified datasets were modelled, resulting in similar parameter estimates. Our model indicated that fosfomycin is likely to be close to 100% excreted via kidneys as the estimate of nonrenal clearance was very low. However, due to the limitation of our study, it is not conclusive, so more research is needed to confirm this finding. Meanwhile, Wenzler et al. evaluated the plasma and urine PK of a single dose of 8 g fosfomycin in healthy volunteers using noncompartmental analyses, resulting in a total clearance of 7.8 L/h and renal clearance of 6.3 L/h [41]. This means that approximately 20% of fosfomycin was eliminated via other routes besides the kidneys. However, the second elimination route was not described in Wenzler’s study. It is known that fosfomycin is not metabolised in the human body and is mainly excreted unchanged in urine through glomerular filtration [43]. In the case of patients with decreased renal function, other routes of fosfomycin elimination may occur, such as biliary excretion, as fosfomycin has been detected in the bile [34, 44, 45]. Still, the understanding of fosfomycin elimination is very limited as the data are scarce. Moreover, the presence of fosfomycin metabolites is hardly studied because there is no validated assay to analyse it.

In conclusion, our simulation suggests that the dose of 4 g every 8 h is probably optimal to treat *E. coli* isolates within the wild-type distribution (ECOFF) with MICs of ≤ 4 g/L for both systemic and urinary tract infections. For the above wild-type MIC and up to the current clinical breakpoint of 32 mg/L, the dosages of 8 g every 8 h and 0.75 g/h may be needed. Although no dosing regimen may be able to kill *E.* coli with MIC above the clinical breakpoint, all simulated dosing regimens can be used to inhibit *E. coli* from reproducing using the AUC_24h_/MIC 25 target. This PK model and simulation results are in line with the current clinical breakpoint. Dosing guidelines based on eGFR calculated with the CKD-EPI formula instead of CrCL by Cockcroft–Gault should be further developed and investigated to treat *E. coli* infections with a more optimal dose.

## Supplementary Information

Below is the link to the electronic supplementary material.

Supplementary file1 (JPG 412 KB)

Supplementary file2 (JPG 172 KB)

Supplementary file3 (DOCX 42 KB)

## Data Availability

The data that support the findings of this study are available from the corresponding author upon reasonable request.
