# Supplementary figures and images for: Population plasma and urine pharmacokinetics and the probability of target attainment of fosfomycin in healthy male volunteers
Source: Eur J Clin Pharmacol. 2023 Apr 15;79(6):775–87. doi: 10.1007/s00228-023-03477-5 (PMC10229474; doi:10.1007/s00228-023-03477-5)

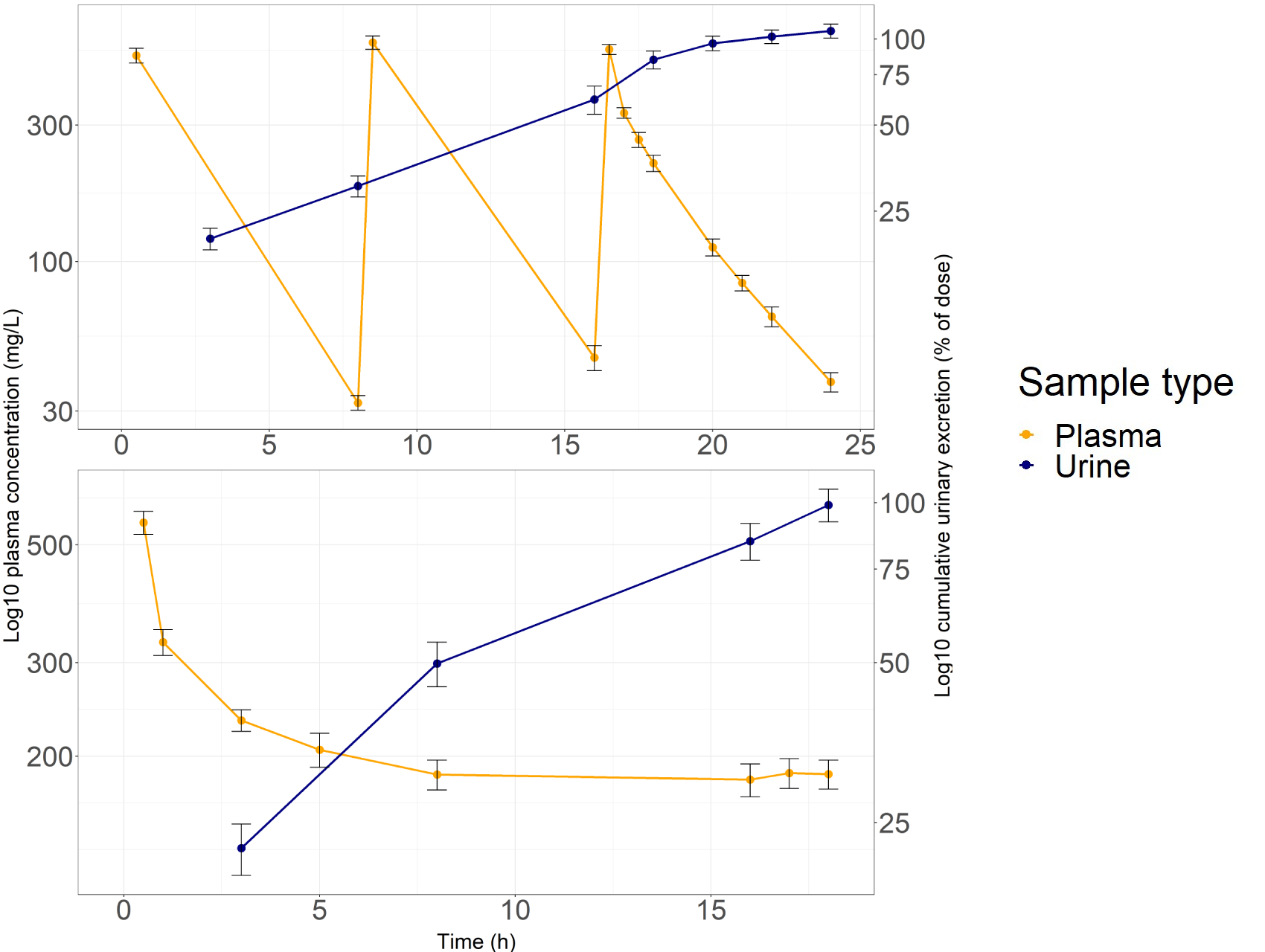

Supplement: Supplementary file 1 — Supplementary file1 (JPG 412 KB) [file 228_2023_3477_MOESM1_ESM.jpg]

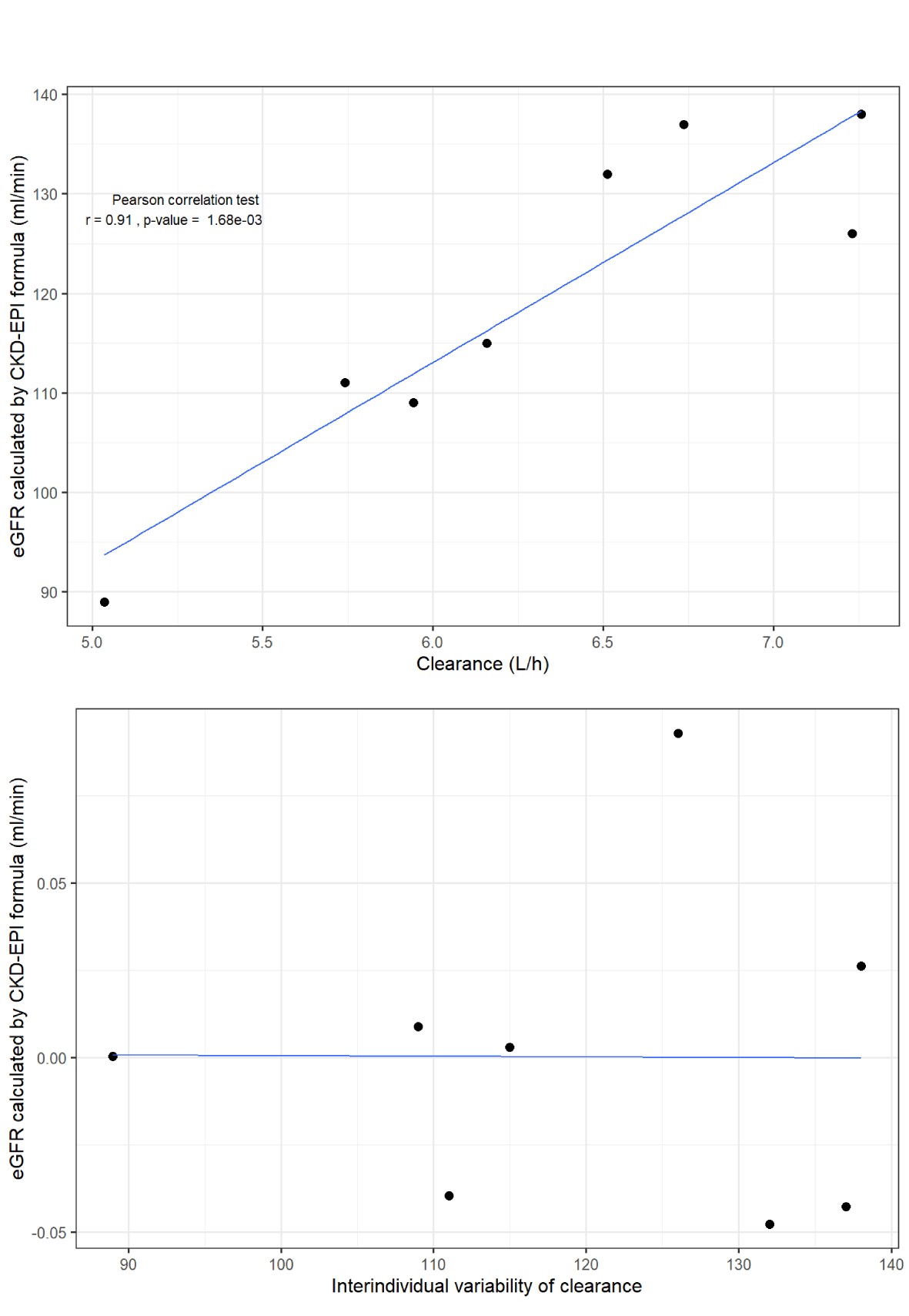

Supplement: Supplementary file 2 — Supplementary file2 (JPG 172 KB) [file 228_2023_3477_MOESM2_ESM.jpg]
