# Supplementary material for: Population plasma and urine pharmacokinetics and the probability of target attainment of fosfomycin in healthy male volunteers
Source: Eur J Clin Pharmacol. 2023 Apr 15;79(6):775–87. doi: 10.1007/s00228-023-03477-5 (PMC10229474; doi:10.1007/s00228-023-03477-5)
Supplement: Supplementary file 3 — Supplementary file3 (DOCX 42 KB) [file 228_2023_3477_MOESM3_ESM.docx]

**Population plasma and urine pharmacokinetics and the probability of target attainment of fosfomycin in healthy male volunteers**

Short running title**: Fosfomycin pharmacokinetic in healthy men**

Angela Elma Edwina (a.e.edwina@gmail.com)^1,2,3^, Birgit C P Koch (b.koch@erasmusmc.nl)^1,2^, Anouk E Muller (a.muller@haaglandenmc.nl)^4,5^, Valentin al Jalali (valentin.aljalali@meduniwien.ac.at)^6^, Peter Matzneller (peter.matzneller@sabes.it)^6,7^, Markus Zeitlinger (markus.zeitlinger@meduniwien.ac.at)^6^, Sebastiaan D T Sassen^1,2*^

^1^Department of Hospital Pharmacy, Erasmus University Medical Center, Rotterdam, Netherlands#, ^2^Rotterdam Clinical Pharmacometrics Group, Rotterdam, The Netherlands#, ^3^Gerontology and Geriatrics Unit, Department of Public Health and Primary care, KU Leuven – University of Leuven, Leuven, Belgium, ^4^Department of Medical Microbiology and Infectious Diseases, Erasmus University Medical Center, Rotterdam, The Netherlands, ^5^Department of Medical Microbiology, Haaglanden Medical Center, The Hague, The Netherlands, ^6^Department of Clinical Pharmacology, Medical University of Vienna, Vienna, Austria, ^7^Service of Rheumatology, Hospital of Merano, South Tyrol Health System ASDAA-SABES, Italy

*Corresponding author: [s.sassen@erasmusmc.nl](mailto:s.sassen@erasmusmc.nl)

#Primary affiliation

# Supplementary Material

NONMEM code for the final model

$PROBLEM PK model

$INPUT ;;

CENSOR ID TIME AMT TAD DV DV=DROP EVID MDV CMT UVOL RATE TRT OCC AGE WEIGHT HEIGHT SCR GFR CRCL BMI ALB PRO

$DATA Plasma_urine_uncensored_2comp_reduced.CSV IGNORE=C

$SUBROUTINES

ADVAN6 TOL=6

$MODEL

COMP=(CENTRAL, DEFDOSE, DEFOBS)

COMP=(PER)

COMP=(URINE)

$PK

COVCLRGFR = (GFR/120)**THETA(7)

TVV1 = THETA(4)

V1 = TVV1

TVCLR = THETA(3)*COVCLRGFR

CLR = TVCLR * EXP(ETA(1))

Q = THETA(5)

V2 = THETA(6)

UVL = UVOL

S1 = V1

S3 = UVL

K12 = Q/V1

K21 = Q/V2

K13 = CLR/V1

$THETA

(0, 0.5) ;1 prop

(0, 1) ;2 prop

(0, 8) ;3 CL

(0, 3) ;4 V1

(0, 10) ;5 Q

(0, 6) ;6 V2

(-10, 1) ;7 GFR

$OMEGA BLOCK(1)

0.03 ; IIV-CLR

$DES

DADT(1) = -K12*A(1)-K13*A(1)+A(2)*K21

DADT(2) = A(1)*K12-A(2)*K21

DADT(3) = A(1)*K13

$SIGMA

1 FIX

1 FIX

$ERROR

IF(CMT.NE.3)THEN

IPRED=F

IRES=DV-IPRED

W1=IPRED*THETA(1)

IWRES = IRES/W1

Y=IPRED + W1*EPS(1)

ELSE

IPRED=F

IRES=DV-IPRED

W2=IPRED*THETA(2)

IWRES = IRES/W2

Y=IPRED + W2*EPS(2)

ENDIF

PLASMA = A(1)

URINE = A(3)

$EST METHOD=1 MAXEVAL=99999 SIG=3 PRINT=5 SADDLE_RESET=1 NOABORT POSTHOC INTERACTION

$COV PRINT=E UNCONDITIONAL

**Supplementary Tables**

**Supplementary table 1.** Probability of target attainment of simulated dosing regimens

|  | Probability AUC_24h_/MIC 98.9 (%) | | | | | | | |
| --- | --- | --- | --- | --- | --- | --- | --- | --- |
| MIC (mg/L) | Intermittent infusion over 30 minutes | | | | | Continuous infusion over 18 h | | |
|  | 4 g/12 h | 4 g/8 h | 4 g/6 h | 8 g/12 h | 8 g/8 h | 0.5 g/h | 0.75 g/h | 1 g/h |
| 0.25 | 100 | 100 | 100 | 100 | 100 | 100 | 100 | 100 |
| 0.5 | 100 | 100 | 100 | 100 | 100 | 100 | 100 | 100 |
| 1 | 100 | 100 | 100 | 100 | 100 | 100 | 100 | 100 |
| 2 | 100 | 100 | 100 | 100 | 100 | 100 | 100 | 100 |
| 4 | 100 | 100 | 100 | 100 | 100 | 100 | 100 | 100 |
| 8 | 100 | 100 | 100 | 100 | 100 | 100 | 100 | 100 |
| 16 | 0 | 99.8 | 99.4 | 100 | 100 | 100 | 100 | 100 |
| 32 | 0 | 0 | 0 | 0 | 99.8 | 0 | 82.8 | 100 |
| 64 | 0 | 0 | 0 | 0 | 0 | 0 | 0 | 0 |
| 128 | 0 | 0 | 0 | 0 | 0 | 0 | 0 | 0 |
| 256 | 0 | 0 | 0 | 0 | 0 | 0 | 0 | 0 |
| 512 | 0 | 0 | 0 | 0 | 0 | 0 | 0 | 0 |
|  | Probability AUC_24h_/MIC 25 | | | | | | | |
| MIC (mg/L) | Intermittent infusion over 30 minutes | | | | | Continuous infusion over 18 h | | |
|  | 4 g/12 h | 4 g/8 h | 4 g/6 h | 8 g/12 h | 8 g/8 h | 0.5 g/h | 0.75 g/h | 1 g/h |
| 0.25 | 100 | 100 | 100 | 100 | 100 | 100 | 100 | 100 |
| 0.5 | 100 | 100 | 100 | 100 | 100 | 100 | 100 | 100 |
| 1 | 100 | 100 | 100 | 100 | 100 | 100 | 100 | 100 |
| 2 | 100 | 100 | 100 | 100 | 100 | 100 | 100 | 100 |
| 4 | 100 | 100 | 100 | 100 | 100 | 100 | 100 | 100 |
| 8 | 100 | 100 | 100 | 100 | 100 | 100 | 100 | 100 |
| 16 | 100 | 100 | 100 | 100 | 100 | 100 | 100 | 100 |
| 32 | 100 | 100 | 100 | 100 | 100 | 100 | 100 | 100 |
| 64 | 0 | 99.6 | 98.3 | 100 | 100 | 100 | 100 | 100 |
| 128 | 0 | 0 | 0 | 0 | 99.6 | 0 | 76.7 | 100 |
| 256 | 0 | 0 | 0 | 0 | 0 | 0 | 0 | 0 |
| 512 | 0 | 0 | 0 | 0 | 0 | 0 | 0 | 0 |
|  | Probability 75%T_>MIC_ | | | | | | | |
| MIC (mg/L) | Intermittent infusion over 30 minutes | | | | | Continuous infusion over 18 h | | |
|  | 4 g/12 h | 4 g/8 h | 4 g/6 h | 8 g/12 h | 8 g/8 h | 0.5 g/h | 0.75 g/h | 1 g/h |
| 0.25 | 100 | 100 | 100 | 100 | 100 | 100 | 100 | 100 |
| 0.5 | 100 | 100 | 100 | 100 | 100 | 100 | 100 | 100 |
| 1 | 100 | 100 | 100 | 100 | 100 | 100 | 100 | 100 |
| 2 | 100 | 100 | 100 | 100 | 100 | 100 | 100 | 100 |
| 4 | 98.6 | 100 | 100 | 100 | 100 | 100 | 100 | 100 |
| 8 | 6.9 | 100 | 100 | 99.9 | 100 | 100 | 100 | 100 |
| 16 | 6.9 | 100 | 100 | 99.9 | 100 | 100 | 100 | 100 |
| 32 | 0 | 54.7 | 100 | 6.9 | 100 | 100 | 100 | 100 |
| 64 | 0 | 0 | 0.1 | 0 | 54.7 | 100 | 100 | 100 |
| 128 | 0 | 0 | 0 | 0 | 0 | 0 | 8.3 | 100 |
| 256 | 0 | 0 | 0 | 0 | 0 | 0 | 0 | 0 |
| 512 | 0 | 0 | 0 | 0 | 0 | 0 | 0 | 0 |

**Supplementary table 2.** Probability of target attainment of different eGFR categories

| Continuous infusion 1 g/h over 18 h | | | | |
| --- | --- | --- | --- | --- |
| MIC (mg/L) | Probability AUC_24h_/MIC 98.9 | | | |
|  | eGFR 90 mL/min | eGFR 110 mL/min | eGFR 120 mL/min | eGFR 140 mL/min |
| 0.25 | 100 | 100 | 100 | 100 |
| 0.5 | 100 | 100 | 100 | 100 |
| 1 | 100 | 100 | 100 | 100 |
| 2 | 100 | 100 | 100 | 100 |
| 4 | 100 | 100 | 100 | 100 |
| 8 | 100 | 100 | 100 | 100 |
| 16 | 100 | 100 | 100 | 100 |
| 32 | 100 | 100 | 100 | 99.6 |
| 64 | 0 | 0 | 0 | 0 |
| 128 | 0 | 0 | 0 | 0 |
| 256 | 0 | 0 | 0 | 0 |
| 512 | 0 | 0 | 0 | 0 |
| MIC (mg/L) | Probability AUC_24h_/MIC 25 | | | |
|  | eGFR 90 mL/min | eGFR 110 mL/min | eGFR 120 mL/min | eGFR 140 mL/min |
| 0.25 | 100 | 100 | 100 | 100 |
| 0.5 | 100 | 100 | 100 | 100 |
| 1 | 100 | 100 | 100 | 100 |
| 2 | 100 | 100 | 100 | 100 |
| 4 | 100 | 100 | 100 | 100 |
| 8 | 100 | 100 | 100 | 100 |
| 16 | 100 | 100 | 100 | 100 |
| 32 | 100 | 100 | 100 | 100 |
| 64 | 100 | 100 | 100 | 100 |
| 128 | 100 | 100 | 100 | 98.8 |
| 256 | 0 | 0 | 0 | 0 |
| 512 | 0 | 0 | 0 | 0 |
| MIC (mg/L) | Probability 75%T_>MIC_ | | | |
|  | eGFR 90 mL/min | eGFR 110 mL/min | eGFR 120 mL/min | eGFR 140 mL/min |
| 0.25 | 100 | 100 | 100 | 100 |
| 0.5 | 100 | 100 | 100 | 100 |
| 1 | 100 | 100 | 100 | 100 |
| 2 | 100 | 100 | 100 | 100 |
| 4 | 100 | 100 | 100 | 100 |
| 8 | 100 | 100 | 100 | 100 |
| 16 | 100 | 100 | 100 | 100 |
| 32 | 100 | 100 | 100 | 100 |
| 64 | 100 | 100 | 100 | 100 |
| 128 | 100 | 100 | 100 | 97.4 |
| 256 | 0 | 0 | 0 | 0 |
| 512 | 0 | 0 | 0 | 0 |

| Intermittent infusion 8g every 8h | | | | |
| --- | --- | --- | --- | --- |
| MIC (mg/L) | Probability AUC_24h_/MIC 98.9 | | | |
|  | eGFR 90 mL/min | eGFR 110 mL/min | eGFR 120 mL/min | eGFR 140 mL/min |
| 0.25 | 100 | 100 | 100 | 100 |
| 0.5 | 100 | 100 | 100 | 100 |
| 1 | 100 | 100 | 100 | 100 |
| 2 | 100 | 100 | 100 | 100 |
| 4 | 100 | 100 | 100 | 100 |
| 8 | 100 | 100 | 100 | 100 |
| 16 | 100 | 100 | 100 | 100 |
| 32 | 100 | 100 | 99.8 | 74.2 |
| 64 | 0 | 0 | 0 | 0 |
| 128 | 0 | 0 | 0 | 0 |
| 256 | 0 | 0 | 0 | 0 |
| 512 | 0 | 0 | 0 | 0 |
| MIC (mg/L) | Probability AUC_24h_/MIC 25 | | | |
|  | eGFR 90 mL/min | eGFR 110 mL/min | eGFR 120 mL/min | eGFR 140 mL/min |
| 0.25 | 100 | 100 | 100 | 100 |
| 0.5 | 100 | 100 | 100 | 100 |
| 1 | 100 | 100 | 100 | 100 |
| 2 | 100 | 100 | 100 | 100 |
| 4 | 100 | 100 | 100 | 100 |
| 8 | 100 | 100 | 100 | 100 |
| 16 | 100 | 100 | 100 | 100 |
| 32 | 100 | 100 | 100 | 100 |
| 64 | 100 | 100 | 100 | 100 |
| 128 | 100 | 100 | 99.6 | 68.1 |
| 256 | 0 | 0 | 0 | 0 |
| 512 | 0 | 0 | 0 | 0 |
| MIC (mg/L) | Probability 75%T_>MIC_ | | | |
|  | eGFR 90 mL/min | eGFR 110 mL/min | eGFR 120 mL/min | eGFR 140 mL/min |
| 0.25 | 100 | 100 | 100 | 100 |
| 0.5 | 100 | 100 | 100 | 100 |
| 1 | 100 | 100 | 100 | 100 |
| 2 | 100 | 100 | 100 | 100 |
| 4 | 100 | 100 | 100 | 100 |
| 8 | 100 | 100 | 100 | 100 |
| 16 | 100 | 100 | 100 | 100 |
| 32 | 100 | 100 | 100 | 100 |
| 64 | 100 | 93.7 | 54.7 | 0.7 |
| 128 | 0 | 0 | 0 | 0 |
| 256 | 0 | 0 | 0 | 0 |
| 512 | 0 | 0 | 0 | 0 |

**Supplementary table 3.** Maximum concentrations following continuous infusion administration

| Subject | Cmax (mg/L) |
| --- | --- |
| 1 | 491.25 |
| 2 | 552.03 |
| 3* | 730.99 |
| 4* | 577.98 |
| 5 | 564.18 |
| 6 | 508.23 |
| 7 | 456.44 |
| 8 | 522.20 |
| mean | 550.41 |

*Thrombophlebitis was occurred

**Supplementary table 4.** Summary of model-building steps of the population pharmacokinetics of fosfomycin

| Model number | Hypothesis | IIV | Error model | ∆OFV | OFV | Condition number |  |
| --- | --- | --- | --- | --- | --- | --- | --- |
| **Structural model** | | | | | | | |
| 1 | One-compartment | On CL | Proportional error |  | 2893.897 | 89 | Two compartment is better than one compartment. Then model 2 was explored further |
| 2 | Two-compartment | On CL | Proportional error | -138.51 | 2755.389 | 526 |  |
| **IIV exploration** | | | | | | | |
| 3 | Two-compartment | On CL and Vc | Proportional error | -169.55 | 2724.342 | 1e^15^ | Two IIVs resulted in high condition number, so model 2 with one IIV was chosen. |
| 4 | Two-compartment | On CL and Vp | Proportional error | -166.75 | 2727.149 | 5.1e^15^ |  |
| 5 | Two-compartment | On CL and Q | Proportional error | -148.77 | 2745.130 | -1.4e^16^ |  |
| **Error model exploration** | | | | | | | |
| 4 | Two-compartment | On CL | Additive error |  | 2878.118 | NA (Boundary problem, cannot estimate Vc) | Model 2 with only proportional errors was chosen as it has a low condition number. |
| 5 | Two-compartment | On CL | Combined error |  | 2740.871 | 1.7e^16^ |  |
|  |  |  |  |  |  |  |  |
| **Covariate analysis** | | | | | | | |
| **Univariate analysis** | |  |  | **Reference model 2** |  |  |  |
| 6 | Albumin influence CL? | On CL | Proportional error | -7.456 | 2747.933 | 5e^11^ | Model with eGFR (CKD-EPI) had the most significant OFV decrease and was chosen as a reference for the forward inclusion. |
| 7 | Age influence CL? | On CL | Proportional error | -0.985 | 2754.404 | 7.8e^8^ |  |
| 8 | Body weight influence CL? | On CL | Proportional error | -2.369 | 2753.02 | 3.2e^7^ |  |
| 9 | BMI influence CL? | On CL | Proportional error | -2.989 | 2752.4 | 4.4e^8^ |  |
| 10 | Protein influence CL? | On CL | Proportional error | -7.471 | 2747.918 | 2.3e^7^ |  |
| 11 | eGFR (CKD-EPI) influence CL? | On CL | Proportional error | -12.909 | 2742.48 | 8.5e^7^ |  |
| 12 | eGFR (CKD-EPI) normalized by BSA influence CL? | On CL | Proportional error | -4.186 | 2751.203 | 3.8e^9^ |  |
| 13 | Serum creatinine influence CL? | On CL | Proportional error | -1.551 | 2753.838 | 2.3e^9^ |  |
| 14 | Clearance creatinine (Cockcroft-Gault) influence CL? | On CL | Proportional error | -5.997 | 2749.392 | 3.9e^6^ |  |
| **Forward inclusion** | |  |  | **Reference model 11** |  |  |  |
| 15 | eGFR (CKD-EPI), albumin | On CL | Proportional error | -3.764 | 2738.716 |  | Model with eGFR (CKD-EPI) and protein (model 16) was chosen as a reference for the backward elimination. |
| 16 | eGFR (CKD-EPI), protein | On CL | Proportional error | -6.124 | 2747.918 |  |  |
| **Backward elimination** | |  |  | **Reference model 16** |  |  |  |
| 17 | Minus protein | On CL | Proportional error | 6.124 | 2742.48 |  | Model with eGFR (CKD-EPI) as covariate (model 17) was chosen as a final model. |
| 18 | Minus GFR | On CL | Proportional error | 11.562 | 2747.918 |  |  |
